# Excessive Pro-Inflammatory Serum Cytokine Concentrations in Virulent Canine Babesiosis

**DOI:** 10.1371/journal.pone.0150113

**Published:** 2016-03-08

**Authors:** Amelia Goddard, Andrew L. Leisewitz, Mads Kjelgaard-Hansen, Annemarie T. Kristensen, Johan P. Schoeman

**Affiliations:** 1 Department of Companion Animal Clinical Studies, Faculty of Veterinary Science, University of Pretoria, Pretoria, South Africa; 2 Department of Veterinary Clinical and Animal Sciences, Faculty of Health and Medical Sciences, University of Copenhagen, Copenhagen, Denmark; CSIRO, AUSTRALIA

## Abstract

*Babesia rossi* infection causes a severe inflammatory response in the dog, which is the result of the balance between pro- and anti-inflammatory cytokine secretion. The aim of this study was to determine whether changes in cytokine concentrations were present in dogs with babesiosis and whether it was associated with disease outcome. Ninety-seven dogs naturally infected with *B*. *rossi* were studied and fifteen healthy dogs were included as controls. Diagnosis of babesiosis was confirmed by polymerase chain reaction and reverse line blot. Blood samples were collected from the jugular vein at admission, prior to any treatment. Cytokine concentrations were assessed using a canine-specific multiplex assay on an automated analyser. Serum concentrations of interleukin (IL)-2, IL-6, IL-8, IL-10, IL-18, granulocyte-macrophage colony stimulating factor (GM-CSF) and monocyte chemotactic protein-1 (MCP-1) were measured. Twelve of the *Babesia*-infected dogs died (12%) and 85 survived (88%). *Babesia*-infected dogs were also divided into those that presented within 48 hours from displaying clinical signs, and those that presented more than 48 hours after displaying clinical signs. Cytokine concentrations were compared between the different groups using the Mann-Whitney *U* test. IL-10 and MCP-1 concentrations were significantly elevated for the *Babesia*-infected dogs compared to the healthy controls. In contrast, the IL-8 concentration was significantly decreased in the *Babesia*-infected dogs compared to the controls. Concentrations of IL-6 and MCP-1 were significantly increased in the non-survivors compared to the survivors. Concentrations for IL-2, IL-6, IL-18 and GM-CSF were significantly higher in those cases that presented during the more acute stage of the disease. These findings suggest that a mixed cytokine response is present in dogs with babesiosis caused by *B*. *rossi*, and that an excessive pro-inflammatory response may result in a poor outcome.

## Introduction

Canine babesiosis caused by *Babesia rossi* is considered the most virulent form of the disease, with a mortality rate in complicated cases of around 10%, of which 80% die within the first 24 hours of admission [1]. There is sufficient evidence that the disease caused by *B*. *rossi* is mediated by an exuberant blood-borne inflammation possibly due to ineffective modulation. This results in organ damage and in some cases death due to organ failure [2–4]. C-reactive protein (CRP) and serum amyloid A (SAA), both considered major acute phase proteins in the dog, have been reported to be significantly increased in canine babesiosis; nevertheless, neither of them demonstrated correlation to severity of disease or outcome [3,5–7]. It has long been speculated that babesiosis and *Plasmodium falciparum* malaria share a common disease process. Both diseases are caused by an intra-erythrocytic protozoan and their pathology is believed to be the result of excessive production of pro-inflammatory cytokines [8,9]. Similarities in the pathology between babesiosis and malaria include severe haemolytic anaemia, icterus, coagulopathies, neurological signs, pulmonary oedema, circulatory collapse and acute kidney damage [4,8]. The fact that both diseases are so similar with regards to clinical signs and pathological lesions may imply that the mediators downstream from the initiating trigger are likely to be the same within each host.

Cytokines and chemokines are a group of endogenous inflammatory and immunomodulating proteins that play a key role in the host response to systemic inflammatory diseases. Chemokines are chemotactic cytokines that play a role in bridging the innate and adaptive immune system by orchestrating the migration of leukocytes and other cells [10–12]. Pro-inflammatory cytokines and chemokines, such as tumour necrosis factor (TNF)-α, interferon (INF)-γ, interleukin (IL)-1, IL-2, IL-6, IL-8, IL-12, IL-18 and monocyte-chemotactic protein-1 (MCP-1) are necessary for initiating an effective inflammatory response [10,11,13]. TNF-α and IL-1β are considered the initiators or proximal cytokines of the pro-inflammatory cytokine cascade in response to infectious disease which results in the production of other cytokines such as IL-6 and IL-8 or distal cytokines [10,14]. This response regulates cellular immune functions and ultimately results in the resolution of the infection. In contrast, inflammation modulating cytokines, such as IL-4, IL-10 and transforming growth factor (TGF)-β, are required to control and down-regulate the cell-mediated inflammatory response by virtue of their ability to suppress the gene expression for pro-inflammatory cytokines [11,15]. An imbalance in host regulation of the pro-inflammatory systemic response and the compensatory modulating response is one of the reasons for systemic inflammatory conditions, such as *P*. *falciparum* malaria and other septic conditions in humans, to progress to multiple organ dysfunction and death in some individuals [14–17].

Similar to babesiosis, the blood stage of the malaria parasite is largely responsible for the pathology associated with the disease. The presence of *P*. *falciparum* organisms within the erythrocytes result in a strong cytokine-mediated inflammatory response by the host once the schizont (stage within the erythrocyte) ruptures [12,18]. Cytokines in malaria are important players in regulating the disease progression and are associated with the appearance of disease symptoms, levels of parasitaemia, disease severity and outcome [12,17,19,20]. An early and effective pro-inflammatory cytokine response is required for the resolution of parasitaemia and control of the malaria infection, balanced with a rapid suppression of this response by modulating cytokines [21]. An excessive pro-inflammatory response, with high concentrations of cytokines such as TNF-α, IFN-γ, IL-6, IL-8, IL-18 and MCP-1 has been associated with severe malaria and death [13,19,20,22–24]. Regulatory cytokines such as IL-10 and TGF-β are important to dampen down this pro-inflammatory response [25].

A multiplex assay enables the simultaneous measurement of multiple cytokines and chemokines in a single sample, which may assist in understanding the host response in virulent canine babesiosis. The objectives of this study were to investigate cytokine concentrations in severe canine babesiosis, caused by *B*. *rossi*, as well as determine whether there was a difference in concentrations between dogs that present early or late during the course of the disease. We hypothesised that, similar to *falciparum* malaria, an excessive pro-inflammatory response exists in severe canine babesiosis, which is more pronounced in dogs that do not survive. In addition, we hypothesised that those dogs that presented later during the course of the disease will have lower cytokine concentrations compared to those dogs that presented earlier.

## Materials and Methods

### Study population

The study population included client-owned dogs, naturally infected with *B*. *rossi* that presented for veterinary care to the Onderstepoort Veterinary Academic Hospital, at the Faculty of Veterinary Science, University of Pretoria between October 2011 and April 2013. The research protocol was approved by the Animal Ethics Committee of the University of Pretoria (Protocol no: V055-11). An initial diagnosis of infection with babesiosis was made through the recognition of commensurate clinical signs and demonstration of intra-erythrocytic trophozoites and merozoites on stained thin blood smears, and was later confirmed as *B*. *rossi* by polymerase chain reaction (PCR) and reverse line blot (RLB).

Dogs of either sex and of any breed were eligible for inclusion in the study provided they were >12 weeks of age, weighed >5 kg, and had a demonstrable parasitaemia. Dogs were excluded if they were subsequently proven by PCR and RLB to be infected with *B*. *vogeli* or *Ehrlichia canis*, or euthanized for reasons other than poor prognosis. Dogs were also excluded if any concurrent inflammatory disease conditions, any known cardiac disease, any known neoplastic disease, any obvious infections or wounds, or any signs of trauma were present. Treatment with any anti-inflammatory medication either at presentation or within 4 weeks prior to presentation was also a reason for exclusion. The number of days sick prior to presentation was recorded for the infected dogs. Dogs received standard care for canine babesiosis, which included antibabesial treatment with diminazene aceturate (Berenil RTU 0.07 g/mL, Intervet, Kempton Park, South Africa) at 3.5 mg/kg, transfusion with packed red cells and intravenous fluids as needed. In addition, any complications were treated accordingly at the discretion of the attending clinician. Outcome was recorded as short-term survival (i.e. until discharge), or death/euthanasia due to poor prognosis.

The control dogs included 15 healthy, client-owned dogs admitted for routine ovariohysterectomy, castration, or blood donation. The control dogs were deemed healthy based on history, a full clinical examination, peripheral blood smear evaluation, complete blood count (CBC), full biochemistry profile, as well as PCR and RLB to rule out infection. Owner consent was obtained for enrolment of all the cases in this study.

### Patient sampling

At presentation and before any treatment, peripheral blood was collected from the jugular vein of each patient and control case with a 21-gauge needle by careful venipuncture. Blood for a CBC was collected into a potassium EDTA vacutainer tube, and for CRP-, SAA- and cytokine concentrations into a serum vacutainer tube. The EDTA sample was also used to perform the PCR and RLB assays. The serum sample was left to clot and then centrifuged at 2100 *g* for 8 minutes and aliquoted into different cryovials. The remainder of the serum sample was stored at -80°C. Serum aliquots were transported on dry ice to the department of Veterinary Clinical and Animal Sciences, University of Copenhagen, Denmark for measurement of CRP-, SAA- and cytokine concentrations, and transit time for the shipment was <48 hours.

### DNA extraction and PCR

DNA was extracted from 200 μL of each EDTA-anticoagulated whole blood sample using a blood and tissue extraction kit (QIAmp blood and tissue extraction kit, Qiagen, Venlo, The Netherlands) according to the manufacturer’s instructions. Molecular diagnosis of *B*. *rossi* and exclusion of other *Babesia*, *Theileria*, *Ehrlichia* and *Anaplasma* species was performed using PCR and RLB. PCR was conducted with a set of primers that amplified a 460–540 base pair fragment of the 18S SSU rRNA spanning the V4 region, a region conserved for *Babesia* and *Theileria*. The *Ehrlichia* PCR amplified the V1 hypervariable region of the 16S SSU rRNA. The membrane used for RLB included probes for *B*. *vogeli*, *B*. *rossi*, *B*. *canis* and *E*. *canis* [26].

### Analyses

CBCs were performed by the Clinical Pathology laboratory, University of Pretoria, South Africa with an automated analyzer (ADVIA 2120, Siemens, Munich, Germany) and data recorded included the white blood cell (WBC) count and differential leukocyte counts that were manually confirmed by an experienced laboratory technologist. Serum CRP-, SAA- and cytokine concentrations were analysed by the department of Veterinary Clinical and Animal Sciences, University of Copenhagen, Denmark. CRP concentrations were measured by an automated commercial human turbidimetric immunoassay (CRP, Randox, Crumlin, UK) using purified canine CRP for calibration (Canine CRP; LifeDiagnostics, Pennsylvania, USA). SAA concentrations were measured by an automated latex agglutination turbidimetric immunoassay (SAA-1 reagent provided by Eiken Chemical Company, Tokyo, Japan). Both parameters were analysed using an automated clinical chemistry analyzer (ADVIA 1800; Siemens, Munich, Germany), and both assays have been validated for use in dogs [27–29].

Serum cytokine concentrations of seven cytokines (IL-2, IL-6, IL-8, IL-10, IL-18, granulocyte-macrophage colony stimulating factor (GM-CSF) and MCP-1) were assessed using a commercially available canine-specific multiplex immunoassay (MILLIPLEX MAP Canine Cytokine/Chemokine Magnetic Bead Panel CCYTO-90K-07, Millipore, Billerica, USA) including internal quality control material with an automated analyser (Luminex 200, Luminex Corporation, Austin, USA) [30]. All cytokines were assayed in duplicate according to the manufacturer’s instructions. Seven-point standard curves, created from included standards of known concentrations of recombinant canine cytokines, were analysed using a five-parameter logistic regression curve-fitting method to determine the concentration of each cytokine. Intra-assay and inter-assay coefficients of variation stated by the manufacturer for all cytokine analytes were <5% and <17% respectively. The minimum detectable concentrations of the seven cytokines provided by the manufacturer were regarded as the detection limits in this study and values below the detection limit were assigned a value equal to the minimum detectable concentration for the respective cytokine.

### Statistical analysis

The infected dogs were divided according to outcome, as well as according to the number of days sick prior to presentation. Statistical analysis was performed using a commercial software package (SPSS Statistics 22.0 ^®^Software; SPSS Inc, StataCorp, College Station, Texas, USA). Data was assessed for normality using the Shapiro Wilk test. The majority of variables were found to be not normally distributed and, therefore, a non-parametric test, the Mann Whitney *U* test, was used to determine significance across groups for each variable. Correlation of cytokine concentration to leukocyte count at admission was assessed using the Spearman’s rank correlation test. Gender proportions between groups were compared using the Chi square test. Data are presented as median and interquartile range (IQR). Level of significance was set at *P* <0.05.

## Results

### Study population characteristics

Ninety-seven dogs naturally infected with *B*. *rossi* and 15 healthy control dogs were included. Twelve *Babesia*-infected dogs died (12%) and 85 dogs survived (88%). Of the infected dogs only 84 had data available on the number of days sick prior to presentation. The median number of days sick prior to presentation was 2 days (48 hours). Forty-four dogs were sick for ≤48 hours and 40 dogs for >48 hours. The abnormalities (seen alone or in various combinations) observed with the non-survivors included: severe anaemia, hyperlactataemia, icterus with raised liver enzymes (>2 times above the highest laboratory reference interval value), hypoglycaemia, hypokalaemia, neurological signs (seizures or coma in the absence of hypoglycaemia), acute respiratory distress due to pulmonary oedema, acute kidney disease, haemoconcentration and secondary immune-mediated haemolytic anaemia (IMHA). There were no significant differences in age and weight between the groups. The ratio of male:female for each group was as follows: controls (5:10), *Babesia*-infected (63:34), survivors (56:29) and non-survivors (7:5) with a significant difference (*P* = 0.02) between the *Babesia*-infected dogs and controls.

### Cytokine, acute phase protein and leukocyte analyses

**Fig 1:**
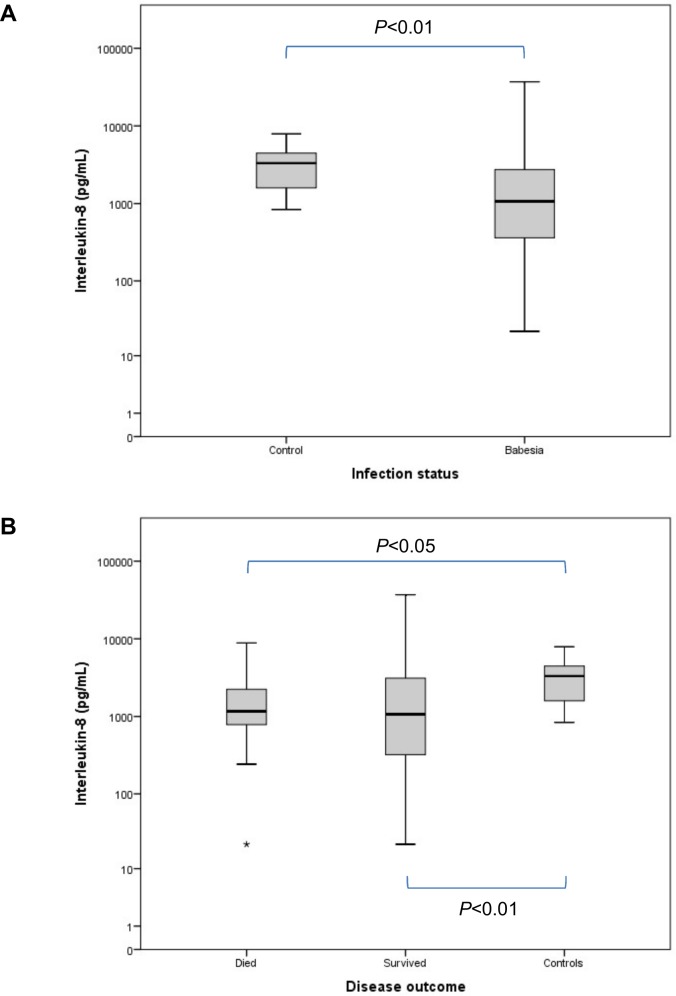
Box plots of the admission IL-8 in the *Babesia*-infected group (A), survivors and non-survivors (B), compared to the healthy control group. The box represents the IQR (i.e. the middle 50% of the observations) with the line inside the box as the median. The whiskers represent the main body of the data, indicating the range of the data. Extreme outlier values that are 3 times removed from the IQR are represented by an asterisk.

**Fig 2:**
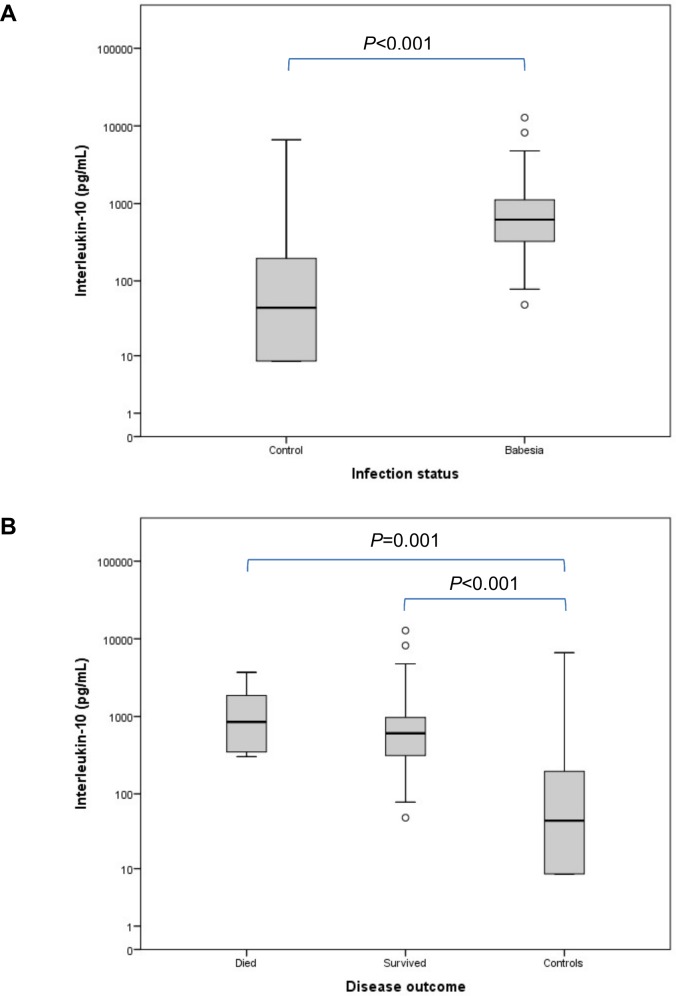
Box plots of the admission IL-10 in the *Babesia*-infected group (A), survivors and non-survivors (B), compared to the healthy control group. The box represents the IQR (i.e. the middle 50% of the observations) with the line inside the box as the median. The whiskers represent the main body of the data, indicating the range of the data. Outliers, values that are 1.5 times removed from the IQR, are plotted as open circles.

**Fig 3:**
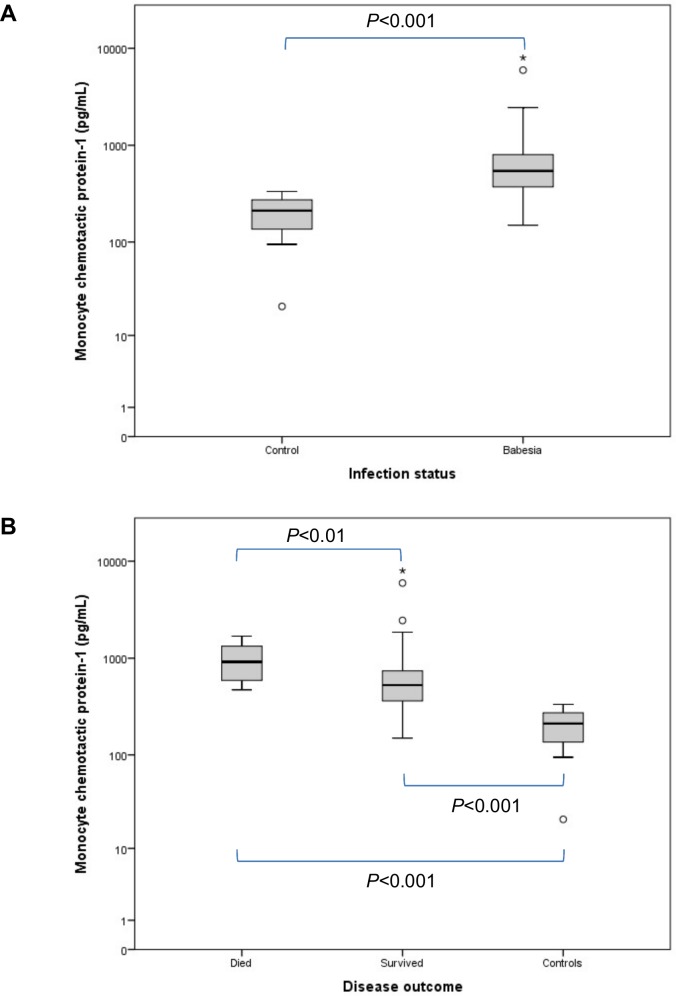
Box plots of the admission MCP-1 in the *Babesia*-infected group (A), survivors and non-survivors (B), compared to the healthy control group. The box represents the IQR (i.e. the middle 50% of the observations) with the line inside the box as the median. The whiskers represent the main body of the data, indicating the range of the data. Outliers, values that are 1.5 times removed from the IQR, are plotted as open circles. Extreme outlier values that are 3 times removed from the IQR are represented by asterisks.

**Fig 4:**
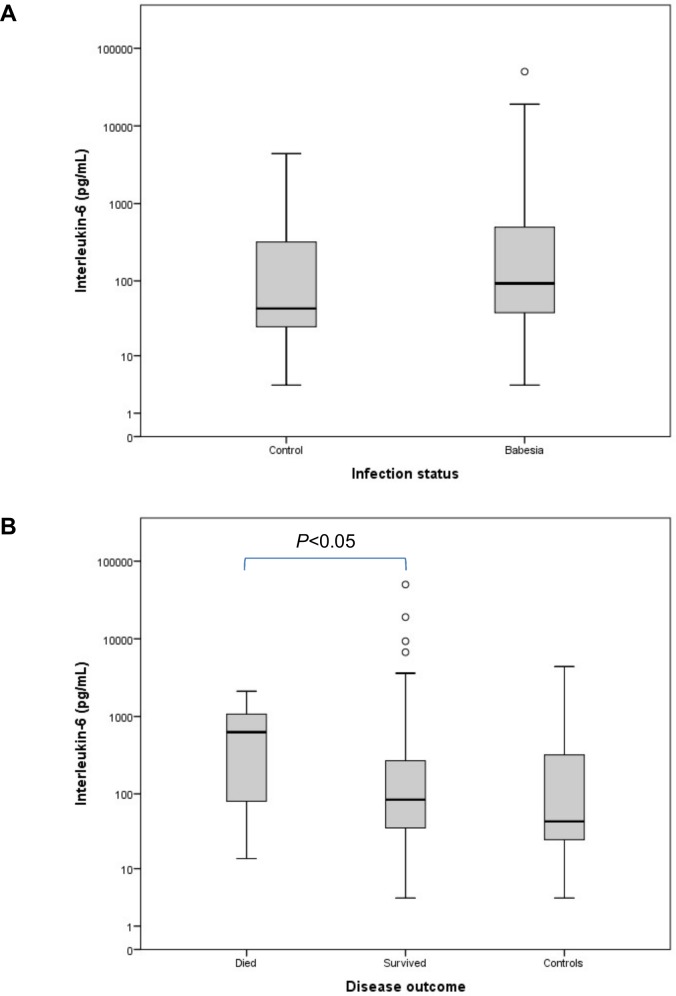
Box plots of the admission IL-6 in the *Babesia*-infected group (A), survivors and non-survivors (B), compared to the healthy control group. The box represents the IQR (i.e. the middle 50% of the observations) with the line inside the box as the median. The whiskers represent the main body of the data, indicating the range of the data. Outliers, values that are 1.5 times removed from the IQR, are plotted as open circles.

Table 1 contains a summary of all the variables for the groups at presentation. Compared to the control group, IL-8 concentration (Fig 1) was significantly decreased in the *Babesia*-infected group (*P* = 0.005). Concentrations of IL-10 (Fig 2) and MCP-1 (Fig 3) were significantly increased, compared to the controls, in the *Babesia*-infected group (*P*<0.001 for both). Only IL-6 (Fig 4) and MCP-1 (Fig 3) concentrations were significantly higher in the non-survivors compared to the survivors (*P* = 0.041 and *P* = 0.009, respectively). IL-2, IL-18 and GM-CSF concentrations were not significantly different between the *Babesia*-infected and control groups, as well as between the survivors and non-survivors. However, concentrations for IL-2, IL-6, IL-18 and GM-CSF were significantly higher in the dogs that were sick for ≤48 hours compared to those that were sick for >48 hours prior to presentation (*P* = 0.001, *P* = 0.009, *P* = 0.001 and *P* = 0.001, respectively).

**Table 1:** Descriptive statistics for leukocyte counts, acute phase protein and cytokine concentrations in dogs with babesiosis and healthy controls.

Analyte	Unit	Controls (n = 15) Median (IQR)	*Babesia*-infected			Survivors (n = 85) Median (IQR)	Non-survivors (n = 12) Median (IQR)
			Total group (n = 97) Median (IQR)	Disease duration prior to presentation ≤48 h (n = 44) Median (IQR)	Disease duration prior to presentation >48 h (n = 40) Median (IQR)		
White blood cell count^a^^,^^c^	×10^9^/L	10.6 (8.1–12.2)	6.2 (4.7–11.2)	5.7 (4.5–6.7)	7.4 (5.8–12.5)	6.2 (4.7–10.5)	6.3 (4.7–17.5)
Segmented neutrophil count^a^^,^^c^	×10^9^/L	5.8 (4.2–7.6)	4.3 (3.1–6.5)	3.6 (2.7–4.8)	4.6 (3.4–9.0)	4.3 (3.0–5.9)	3.9 (3.2–11.2)
Lymphocyte count^a^	×10^9^/L	2.1 (1.7–2.9)	1.3 (0.9–2.1)	1.3 (0.7–1.7)	1.4 (0.9–2.2)	1.3 (0.9–2.1)	1.4 (0.8–2.4)
Monocyte count	×10^9^/L	0.5 (0.3–1.1)	0.5 (0.3–0.9)	0.5 (0.2–0.7)	0.5 (0.3–1.1)	0.5 (0.3–0.8)	0.8 (0.4–1.9)
Eosinophil count^a^	×10^9^/L	0.7 (0.5–1.3)	0.0 (0.0–0.1)	0 (0–0.1)	0 (0–0.1)	0.0 (0.0–0.1)	0.0 (0.0)
C-Reactive Protein^a^^,^^c^	mg/L	2.6 (2.1–3.8)	111.3 (87.4–154.4)	120.0 (99.1–183.6)	100.2 (78.4–127.5)	111.3 (82.6–154.4)	116.1 (94.3–178.1)
Serum Amyloid A^a^	mg/L	14.1 (11.1–15.3)	933.4 (445.5–1610.0)	1182.1 (591.7–1568.9)	743.0 (428.5–1596.2)	813.7 (434.1–1564.4)	1350.6 (647.8–1896.8)
Interleukin-2^c^	pg/mL	19.0 (12.2–227.3)	28.2 (3.4–107.9)	65.7 (23.5–239.7)	16.0 (3.4–36.1)	27.5 (3.4–100.7)	40.8 (3.4–314.3)
Interleukin-6^b^^,^^c^	pg/mL	43.6 (23.4–348.3)	92.9 (37.1–497.8)	149.1 (66.6–792.5)	57.8 (28.5–206.5)	84.1 (35.7–329.9)	643.9 (77.1–1097.4)
Interleukin-8^a^	pg/mL	3316.7 (1528.1–4886.1)	1066.3 (342.1–2883.5)	836.4 (332.0–1893.6)	1249.2 (477.4–3906.8)	1066.3 (318.0–3209.2)	1171.7 (780.1–2447.8)
Interleukin-10^a^	pg/mL	44.6 (8.4–280.9)	620.3 (319.5–1126.1)	803.6 (340.4–1650.0)	451.2 (304.9–868.1)	607.5 (308.7–1043.5)	849.3 (339.6–2035.1)
Interleukin-18^c^	pg/mL	43.6 (24.8–420.1)	52.3 (30.6–179.1)	120.4 (42.6–452.4)	34.4 (23.2–64.4)	52.3 (31.4–168.1)	65.1 (20.7–610.0)
Granulocyte-macrophage colony stimulating factor^c^	pg/mL	21.8 (9.1–166.3)	29.3 (11.1–130.1)	94.4 (19.5–286.5)	19.8 (9.1–39.8)	29.2 (13.5–123.5)	45.8 (9.1–419.9)
Monocyte chemotactic protein-1^a^^,^^b^	pg/mL	211.7 (115.0–275.3)	544.2 (370.2–813.3)	591.4 (432.1–1267.5)	539.6 (332.8–785.8)	527.6 (361.7–741.2)	921.7 (577.5–1356.2)

^a^ Significance between control and *Babesia*-infected groups (*P*<0.05)

^b^ Significance between survivor and non-survivor groups (*P*<0.05)

^c^ Significance between sick≤48 hours and sick>48 hours (*P*<0.05)

Compared to the control group, the CRP- and SAA concentrations were significantly increased in the *Babesia*-infected group (*P*<0.001). There were no significant differences between the survivors and non-survivors for both. Compared to the controls, the WBC-, segmented neutrophil-, lymphocyte- and eosinophil counts were significantly decreased in the *Babesia*-infected group (*P* = 0.009, *P* = 0.045, *P* = 0.004 and *P*<0.001, respectively). There were no significant differences between the survivor and non-survivor groups. The CRP concentration was significantly higher (*P* = 0.03) and the WBC- and segmented neutrophil counts significantly lower in the dogs that were sick for ≤48 hours compared to those that were sick for >48 hours prior to presentation (*P* = 0.004 and *P* = 0.016, respectively).

For the *Babesia*-infected dogs, IL-8 concentration had weak, yet significant positive correlations with WBC (*r* = 0.303; *P* = 0.003), segmented neutrophil (*r* = 0.25; *P* = 0.014) and lymphocyte (*r* = 0.282; *P* = 0.005) counts. IL-10 concentration had weak, yet significant negative correlations with WBC (*r* = -0.408; *P*<0.001), segmented neutrophil (*r* = -0.364; *P*<0.001) and monocyte (*r* = -0.276; *P* = 0.006) counts. MCP-1 concentration had a weak, yet significant negative correlation with the eosinophil (*r* = -0.371; *P*<0.001) count.

## Discussion

This is the first report on the cytokine profile present in dogs infected with *B*. *rossi* and demonstrates that various key cytokines, such as IL-6, IL-8, IL-10 and MCP-1, were significantly increased or decreased in dogs with *B*. *rossi* infection compared to healthy controls. The degree of increase for some of these cytokines was associated with a poor outcome. Similar increases in cytokine concentrations have been reported in other studies on canine piroplasmoses, such as *B*. *canis* [31,32], *B*. *gibsoni* [7] and *Rangelia vitalli* [33].

Because of the highly inflammatory nature of canine babesiosis [2,3], increased concentrations of the pro-inflammatory cytokine IL-6 would be expected as has been described in humans and dogs with sepsis [34,35], as well as dogs with piroplasmoses [32,33]. In our study high concentrations for IL-6 were recorded in the *Babesia*-infected dogs, but the lack of significance between the *Babesia*-infected dogs and controls could be a result of the wide range present within each group and the substantial overlap between the ranges. Alternatively, it may also be explained by the fact that IL-6 increases early in the course of the disease, as was seen by the significantly higher concentrations in the dogs that presented within 48 hours from displaying clinical signs, and may have already been replaced by other cytokines in those dogs that presented later on in the course of the disease. However, IL-6 was significantly higher in the *Babesia*-infected dogs that died compared to those that survived. IL-6 plays a central role in the host defence during inflammation caused by infections and has a variety of biological effects, including induction of acute phase protein production, such as CRP and SAA, by the hepatocytes, stimulation of adrenocorticotropic hormone, as well as secretion of cortisol, suppression of the hypothalamic-pituitary-thyroid axis, activation of B- and T-lymphocytes, and modulation of haematopoiesis [10,32,36]. In our study concentrations for CRP and SAA were significantly increased in the *Babesia*-infected group, indicating that IL-6 is associated with an inflammatory state in babesiosis, despite the fact that concentrations were not significantly different from the control dogs. The CRP concentration was also significantly higher in those dogs that presented earlier in the course of the disease. In humans, low serum CRP concentration can be used as a hallmark to check for blocked IL-6 activity *in vivo* as a result of an increased concentration of free anti-IL-6 receptor antibody [37]. A previous study reported on high serum cortisol and low thyroxine (T4) concentrations in dogs infected with *B*. *rossi* which were associated with disease severity. The authors of this study suggested that IL-6 may be instrumental in the endocrine changes observed [38]. A recent study showed a strong negative correlation between IL-6 and triiodothyronine (T3) in dogs infected with *B*. *canis* [32]. A lack of consistently high concentrations of IL-6 has been reported in other inflammatory diseases in dogs such as IMHA, pyometra and cervical spondylomyelopathy [30,39,40]. Possible reasons for low concentrations of IL-6 in inflammatory disease include decreased analytical sensitivity, rapid transient increases that evade detection or, in cases that have been sick for a longer period of time, IL-6 may have already been replaced by other cytokines as the main cytokine as may have been the case in our study [30,34].

In contrast, the concentration of IL-8 (or CXCL-8) was significantly decreased in the *Babesia*-infected dogs compared to healthy dogs. This was a surprising finding because elevated serum concentrations for IL-8 have been documented in many inflammatory conditions for both humans and animals [41,42]. Increased IL-8 concentrations are associated with severe malaria and have been shown to correlate with parasite density [12,43]. The primary functions of IL-8, a chemokine, include chemoattraction of neutrophils to sites of inflammation, activation and degranulation of neutrophils and basophils, as well as inhibiting leukocyte adhesion to cytokine-activated endothelial cells [10,15,42,44]. Neutrophils have also been shown to in turn produce IL-8 resulting in perpetuation of the inflammation [45,46]. There were several dogs in the *Babesia*-infected group that had serum concentrations less than the analyser detection limit for IL-8 which was set as 21.7 pg/mL. This could mean that for our study, either the peak serum concentration was missed or that canine babesiosis does not result in an increased circulating IL-8 concentration. A somewhat similar finding was reported in dogs with sepsis where admission IL-8 concentrations were well within the normal reference range with no significant difference compared to healthy controls [47]. However, the low serum concentration did correlate with the significantly lower circulating neutrophil counts that were present in the dogs with babesiosis. Thus, this apparent downregulation of IL-8 expression and accompanying neutropaenia in virulent canine babesiosis deserves further investigation.

IL-10, an important modulating cytokine, was significantly increased in the *Babesia*-infected dogs but with no significant difference between survivors and non-survivors. IL-10 is considered a major modulator of the immune response and inflammatory activity through induction of regulatory T cells and blocking of the production of pro-inflammatory cytokines such as TNF-α, IL-1β, IL-6, IL-8, IL-12 and IL-18 [10,16,48–50]. In addition, IL-10 appears to have the ability to mediate immunostimulatory responses through decreased suppression or increased production of B-cell activating functions [51]. It has also been reported to stimulate neutrophils to synthesise the modulating cytokine IL-1ra, which in turn blocks IL-1β, a potent pro-inflammatory cytokine [52]. In humans with sepsis, IL-10 concentration has been reported to parallel the excessive production of pro-inflammatory cytokines and sustained overproduction of IL-10 has been associated with poor prognosis, most likely due to the development of a state of immunosuppression [11]. Similarly, in humans with early-stage systemic inflammatory response, a modulating cytokine profile characterised by a high ratio of IL-10 to TNF-α, seems to be associated with a fatal outcome [53]. High IL-10 concentrations have a positive association with *P*. *falciparum* parasitaemia and, therefore, the reported immunosuppression may facilitate parasite persistence [25,52]. Contrary to these findings, severe anaemia in *falciparum* malaria has been associated with high concentrations of TNF-α and low concentrations of IL-10, with the ratio between them being the clearest indicator of disease severity. This reported relative deficiency in IL-10 in fatal malaria cases possibly reflect an inadequate modulating response to an overwhelming pro-inflammatory cytokine production [18,22]. Thus, the role of IL-10 in malaria, and possibly in babesiosis, remains controversial because high concentrations have been shown to be associated with both severe disease and protection against *P*. *falciparum* infections [54].

MCP-1 (or CCL2) was significantly increased in *Babesia*-infected dogs and the degree of increase was associated with a poor outcome. MCP-1 is a pro-inflammatory chemokine and a major mediator of monocyte recruitment from the bone marrow in response to inflammation, and trafficking to sites of inflammation [30,55,56]. Recruitment of monocytes/macrophages in response to inflammation is a vital response to eliminate invading pathogens through phagocytosis [56]. It seems to play an important immunomodulatory role in controlling the balance between pro- and anti-inflammatory cytokines [57]. In humans with sepsis, MCP-1 concentration showed the best correlation with organ dysfunction and mortality compared to numerous other cytokines. MCP-1 was also significantly increased in critically ill dogs [55], IMHA [30], myxomatous mitral valve disease [58] and various cancers [59,60]. It was also identified as a prognostic indicator of outcome in dogs with IMHA and lymphoma [30,60]. The absence of a monocytosis associated with high MCP-1 concentrations may be the result of either a relatively greater effect of MCP-1 on recruitment of monocytes into the tissue than on mobilising monocytes from bone marrow, or the multifactorial nature of monocyte homeostasis at the level of the bone marrow, blood and tissue [60].

There were no significant differences seen between the controls and *Babesia*-infected dogs, including survivors and non-survivors, for IL-2, IL-18 and GM-CSF. Despite this finding, all three cytokines had significantly higher concentrations in those *Babesia*-infected dogs that presented earlier in the course of the disease and are thus more associated with the acute stage of babesiosis. A significant increase in IL-18 concentration has been observed in the acute and recovery phase of uncomplicated *falciparum* malaria, and was correlated with disease severity [61]. The marked lymphopaenia and eosinopaenia present in the infected dogs was most likely due to high concentrations of endogenous glucocorticoids that have been reported to be present in canine babesiosis [38].

Our study had some limitations. Different clinicians managed cases resulting in different treatment preferences, which could have affected the case outcome. We were limited to those cytokines available in the canine-specific kit for the multiplex technology. Cytokines, such as TNF and INF-γ, are considered initiators of inflammation but were not included in the kit. In addition, various limitations are associated with cytokine measurement obtained through immunoassays. For example, timing of sampling, duration of the stimulus for synthesis and duration of release will influence the concentrations of cytokines in the blood. This did have an effect on our study since the *Babesia*-infected cases that presented earlier during the course of the disease did have significantly higher concentrations for some cytokines. The cytokines detected may also not be biologically active, and concentrations in the blood do not necessarily reflect events at the local cellular level [18]. The lack of significance for some variables could have been due to the fact that the number of cases in the non-survivor group was small.

## Conclusions

A complex network of inflammatory and modulating cytokines exists in dogs infected with *B*. *rossi*. Only IL-6 and MCP-1 were identified as markers of a poor outcome in *B*. *rossi* infected dogs and suggests that an excessive pro-inflammatory host response may be an important factor to consider in understanding the underlying cause of death in this condition.

## Supporting Information

S1 File Cytokine Concentrations in Virulent Canine Babesiosis—Leukocyte Data. (PDF)

S2 File Cytokine Concentrations in Virulent Canine Babesiosis—APP and Cytokine Data. (PDF)
