# Supplementary material for: Excessive Pro-Inflammatory Serum Cytokine Concentrations in Virulent Canine Babesiosis
Source: PLoS One. 2016 Mar 8;11(3):e0150113. doi: 10.1371/journal.pone.0150113 (PMC4783066; doi:10.1371/journal.pone.0150113)
Supplement: S1 File — (PDF) [file pone.0150113.s001.pdf]

Case no	Outcome	WBC (x10^9/L)	NEUT (x10^9/L)	LYMPH (x10^9/L)	MONO (x10^9/L)	EOS (x10^9/L)
Case1	Survived	4.83	3.14	0.72	0.53	0.00
Case2	Survived	16.33	10.45	2.61	0.82	0.16
Case3	Survived	5.64	4.40	0.73	0.17	0.00
Case4	Survived	4.11	3.04	0.66	0.33	0.00
Case5	Survived	4.46	2.99	1.29	0.13	0.00
Case6	Survived	17.61	12.33	2.11	1.58	0.00
Case7	Survived	11.09	5.77	3.44	0.55	1.11
Case8	Survived	9.97	5.75	2.51	0.54	0.18
Case9	Survived	5.82	3.72	1.46	0.41	0.06
Case10	Survived	5.27	2.33	1.10	0.53	0.00
Case11	Survived	17.81	9.59	1.67	1.67	0.00
Case12	Survived	6.63	4.77	1.19	0.60	0.00
Case13	Died	16.64	9.48	3.49	2.00	0.00
Case14	Survived	21.06	17.06	2.11	1.26	0.21
Case15	Survived	6.66	4.33	1.73	0.47	0.00
Case16	Survived	7.79	6.15	0.86	0.47	0.00
Case17	Survived	6.36	3.24	2.61	0.19	0.13
Case18	Survived	3.31	1.39	0.75	0.41	0.17
Case19	Survived	8.54	4.36	0.94	0.68	0.00
Case20	Survived	7.87	4.09	2.83	0.55	0.08
Case21	Died	5.78	3.47	1.39	0.35	0.12
Case22	Survived	12.91	8.53	0.79	0.90	0.00
Case23	Survived	3.73	2.54	0.52	0.15	0.07
Case24	Survived	19.73	10.62	2.32	1.66	0.17
Case25	Survived	7.44	2.98	3.42	0.97	0.07
Case26	Survived	7.38	4.72	2.29	0.30	0.00
Case27	Survived	4.63	2.64	1.44	0.42	0.00
Case28	Survived	5.19	3.32	0.99	0.31	0.31
Case29	Survived	5.90	4.78	0.47	0.53	0.06
Case30	Survived	4.29	1.89	0.77	0.94	0.00
Case31	Survived	5.78	3.76	0.87	0.92	0.00
Case32	Survived	6.07	4.49	0.91	0.49	0.00
Case33	Survived	6.08	3.71	1.52	0.61	0.24
Case34	Died	7.40	4.04	1.35	0.81	0.00
Case35	Survived	16.10	4.70	1.18	0.81	0.00
Case36	Survived	5.85	2.77	0.80	0.32	0.05
Case37	Died	23.83	11.02	0.89	2.49	0.00
Case38	Survived	5.60	3.47	0.73	0.90	0.00

Case no	Outcome	WBC (x10^9/L)	NEUT (x10^9/L)	LYMPH (x10^9/L)	MONO (x10^9/L)	EOS (x10^9/L)
Case39	Survived	5.91	5.44	0.12	0.24	0.00
Case40	Survived	5.19	3.22	0.99	0.78	0.05
Case41	Survived	3.68	2.28	0.81	0.33	0.00
Case42	Survived	5.91	4.43	1.00	0.24	0.24
Case43	Survived	22.06	4.25	2.99	0.34	0.00
Case44	Survived	6.22	4.48	1.24	0.31	0.06
Case45	Survived	6.21	4.04	1.37	0.37	0.06
Case46	Survived	6.43	4.31	1.41	0.32	0.06
Case47	Survived	3.06	1.53	0.86	0.55	0.00
Case48	Survived	4.48	1.06	0.76	0.53	0.00
Case49	Survived	12.88	11.08	0.39	0.52	0.00
Case50	Survived	33.12	20.53	4.64	5.30	0.00
Case51	Survived	5.18	3.06	1.50	0.21	0.00
Case52	Survived	11.36	9.09	1.70	0.34	0.11
Case53	Survived	15.58	7.11	2.60	1.50	0.14
Case54	Survived	8.25	4.95	1.57	1.16	0.00
Case55	Died	7.70	3.14	0.81	0.35	0.05
Case56	Survived	10.01	7.01	1.30	0.50	0.20
Case57	Survived	6.16	4.74	1.05	0.31	0.00
Case58	Died	14.75	11.21	0.44	1.33	0.00
Case59	Died	4.62	3.42	0.88	0.14	0.00
Case60	Survived	2.30	1.59	0.35	0.30	0.00
Case61	Died	30.87	16.99	3.35	1.68	0.00
Case62	Survived	24.67	17.27	2.71	2.22	0.00
Case63	Survived	4.75	3.14	1.24	0.38	0.00
Case64	Survived	20.49	10.86	5.12	1.84	0.41
Case65	Died	31.88	16.96	2.57	4.37	0.00
Case66	Survived	3.86	2.70	0.31	0.62	0.00
Case67	Survived	5.38	0.14	1.04	0.40	0.00
Case68	Survived	9.78	5.77	2.93	0.29	0.00
Case69	Survived	6.21	3.35	2.17	0.12	0.00
Case70	Survived	7.35	4.63	1.76	0.29	0.15
Case71	Survived	7.85	5.57	1.65	0.31	0.00
Case72	Survived	16.80	12.26	2.86	1.18	0.17
Case73	Survived	5.87	3.52	1.64	0.53	0.00
Case74	Survived	4.37	2.84	1.09	0.26	0.17
Case75	Survived	12.73	6.87	4.20	0.76	0.25
Case76	Survived	6.34	3.49	2.03	0.44	0.06

Case no	Outcome	WBC (x10^9/L)	NEUT (x10^9/L)	LYMPH (x10^9/L)	MONO (x10^9/L)	EOS (x10^9/L)
Case77	Survived	36.00	24.48	3.96	1.80	0.00
Case78	Survived	7.14	5.14	1.14	0.86	0.00
Case79	Survived	4.69	2.81	0.66	0.75	0.00
Case80	Survived	6.29	3.96	1.70	0.63	0.00
Case81	Died	5.25	1.94	1.55	0.09	0.00
Case82	Survived	4.75	3.15	0.73	0.04	0.04
Case83	Survived	26.35	13.55	1.15	1.15	0.00
Case84	Died	5.19	0.91	1.73	0.49	0.00
Case85	Died	5.49	3.79	0.05	0.80	0.00
Case86	Survived	5.69	4.04	0.34	1.19	0.00
Case87	Survived	13.08	8.24	2.75	0.52	0.52
Case88	Survived	3.34	2.47	0.40	0.27	0.00
Case89	Survived	6.38	4.72	0.89	0.70	0.00
Case90	Survived	5.56	3.06	1.22	0.28	0.22
Case91	Survived	4.72	2.55	1.51	0.09	0.09
Case92	Survived	3.37	0.92	1.59	0.31	0.00
Case93	Survived	13.95	6.00	2.83	1.09	0.00
Case94	Survived	13.94	7.81	3.76	1.81	0.28
Case95	Survived	3.62	2.24	1.19	0.11	0.07
Case96	Survived	3.58	1.00	2.00	0.43	0.00
Case97	Survived	6.67	4.54	1.33	0.67	0.00
Control1		14.07	9.29	3.38	0.14	1.27
Control2		8.58	5.83	1.80	0.69	0.26
Control3		7.80	4.91	1.48	0.23	1.17
Control4		12.22	8.55	2.08	0.61	0.73
Control5		11.60	5.80	2.90	1.28	1.39
Control6		8.05	5.31	1.69	0.32	0.64
Control7		8.99	4.14	2.88	1.26	0.72
Control8		10.57	7.50	1.80	0.74	0.42
Control9		4.72	3.12	0.94	0.38	0.28
Control10		10.84	7.59	2.06	0.54	0.54
Control11		7.86	3.54	2.52	0.39	1.18
Control12		17.23	8.96	1.38	4.31	2.24
Control13		13.28	6.11	5.58	1.06	0.53
Control14		8.39	4.20	2.68	0.42	1.01
Control15		11.46	5.50	2.41	0.23	3.32
