# Supplementary material for: Excessive Pro-Inflammatory Serum Cytokine Concentrations in Virulent Canine Babesiosis
Source: PLoS One. 2016 Mar 8;11(3):e0150113. doi: 10.1371/journal.pone.0150113 (PMC4783066; doi:10.1371/journal.pone.0150113)
Supplement: S2 File — (PDF) [file pone.0150113.s002.pdf]

Case no	Outcome	SAA (mg/L)	CRP (mg/L)	IL-2 (pg/mL)	IL-6 (pg/mL)	IL-8 (pg/mL)	IL-10 (pg/mL)	IL-18 (pg/mL)	GM-CSF (pg/mL)	MCP-1 (pg/mL)
Case1	Survived	1050.8	90.9	20.3	53.5	79.5	532.2	57.0	27.5	791.4
Case2	Survived	803.3	137.4	31.9	62.9	6186.0	244.1	66.3	29.2	314.5
Case3	Survived	1253.7	191.0	617.3	569.1	1393.2	437.3	1766.6	492.9	667.0
Case4	Survived	1311.1	162.8	3.4	28.1	125.7	4762.5	32.4	15.6	315.5
Case5	Survived	601.4	223.9	69.7	138.4	45.1	1167.5	339.1	133.4	519.8
Case6	Survived	2357.6	99.0	13.1	26.5	1310.4	391.5	29.9	13.9	348.5
Case7	Survived	132.7	118.3	12.2	21.8	687.2	1833.6	48.7	18.2	198.9
Case8	Survived	933.4	143.5	29.2	121.1	133.4	370.2	129.5	61.0	673.0
Case9	Survived	480.4	115.9	3.4	28.1	21.6	4756.6	22.3	9.1	424.1
Case10	Survived	1248.1	99.5	92.4	128.9	141.1	1535.9	231.9	159.8	527.6
Case11	Survived	1266.6	99.0	22.0	133.7	5488.8	332.5	48.7	25.0	293.9
Case12	Survived	697.3	116.3	3.4	29.7	8748.6	432.4	17.3	9.1	262.6
Case13	Died	1501.0	215.1	3.4	25.0	21.6	951.5	5.7	9.1	620.7
Case14	Survived	945.9	172.6	3.4	39.3	3561.9	334.2	18.5	9.1	198.2
Case15	Survived	1695.2	50.5	45.2	171.4	2404.1	8189.7	169.3	110.4	613.6
Case16	Survived	2505.1	198.2	2529.7	9315.4	292.9	437.3	745.1	1039.9	1854.0
Case17	Survived	70.9	89.6	135.9	268.3	3376.8	381.7	163.4	85.3	415.9
Case18	Survived	74.3	65.0	34.5	56.6	3117.8	1134.8	86.2	19.0	358.5
Case19	Survived	3001.0	184.8	34.5	94.4	57.3	811.8	52.3	40.3	742.1
Case20	Survived	131.7	72.7	74.2	165.1	2313.8	128.7	124.8	112.4	293.5
Case21	Died	293.1	88.1	53.4	86.5	791.8	1590.3	82.7	62.3	657.1
Case22	Survived	2020.5	120.1	19.4	55.1	2577.1	162.7	43.7	20.7	211.4
Case23	Survived	628.5	71.6	2389.8	6718.3	5639.0	12786.5	2249.8	2920.8	5950.5
Case24	Survived	237.6	56.6	53.4	64.5	10065.4	628.8	97.9	75.9	307.0
Case25	Survived	162.7	77.9	3.4	18.6	857.8	412.8	16.0	9.1	769.0
Case26	Survived	134.0	41.5	3.4	29.7	1964.7	969.7	32.4	9.1	800.2
Case27	Survived	169.0	73.7	136.8	241.8	834.7	851.2	110.8	119.9	330.1
Case28	Survived	451.6	68.4	273.2	495.8	171.9	3019.6	306.1	204.4	459.5
Case29	Survived	1164.0	103.6	37.2	99.1	21.6	555.2	31.2	43.7	563.7
Case30	Survived	291.7	143.9	39.9	77.1	1097.2	607.5	67.5	54.6	372.3
Case31	Survived	650.3	106.0	3.4	3.6	880.8	906.2	24.8	9.1	323.7
Case32	Survived	445.1	180.8	84.2	121.1	3610.1	470.0	166.9	75.9	555.9
Case33	Survived	376.3	30.6	3.4	3.6	256.7	781.8	24.8	9.1	203.3
Case34	Died	962.4	103.3	363.9	1925.9	2737.8	3702.9	981.2	640.5	1665.1
Case35	Survived	679.7	155.9	15.9	73.2	4610.1	961.2	36.6	9.1	527.4
Case36	Survived	2746.3	122.5	164.7	218.2	447.3	228.5	587.9	263.8	544.2
Case37	Died	817.6	91.3	3.4	788.0	2627.9	303.5	5.7	9.1	500.0
Case38	Survived	1255.2	191.8	14.0	19.9	754.1	93.2	45.1	20.3	230.8
Case39	Survived	813.7	228.4	103.4	229.1	111.4	512.8	142.1	117.6	700.6
Case40	Survived	355.9	94.0	3.4	3.6	5693.8	197.4	22.7	9.1	322.9
Case41	Survived	1652.4	59.7	43.3	57.4	101.0	169.7	80.6	44.3	360.7
Case42	Survived	1018.7	118.7	3.4	3.6	686.4	938.4	33.1	9.1	485.0
Case43	Survived	1757.9	129.1	15.0	540.6	9608.0	97.3	43.6	18.5	687.8
Case44	Survived	3017.1	144.9	26.9	143.4	1066.3	889.4	49.5	22.0	1484.3
Case45	Survived	728.0	148.1	3.4	3.6	50.1	140.8	31.4	9.1	327.5
Case46	Survived	3001.0	187.3	261.5	520.5	838.1	276.1	396.1	296.3	459.4
Case47	Survived	548.5	125.0	3.4	29.6	60.5	1799.5	31.4	9.1	402.1
Case48	Survived	256.5	283.1	16.0	94.1	229.1	2191.9	35.7	17.9	1470.9

Case no	Outcome	SAA (mg/L)	CRP (mg/L)	IL-2 (pg/mL)	IL-6 (pg/mL)	IL-8 (pg/mL)	IL-10 (pg/mL)	IL-18 (pg/mL)	GM-CSF (pg/mL)	MCP-1 (pg/mL)
Case49	Survived	1044.2	193.1	3.4	46.2	1188.0	325.5	22.7	9.1	883.9
Case50	Survived	1165.8	89.0	766.1	549.5	1054.8	656.7	1097.0	410.1	535.0
Case51	Survived	1399.7	109.7	98.0	159.7	1851.5	78.0	97.8	94.9	539.8
Case52	Survived	423.0	86.6	17.6	35.7	859.5	176.5	28.8	19.8	651.8
Case53	Survived	757.9	35.8	36.6	74.0	300.8	150.1	25.3	26.7	395.4
Case54	Survived	1861.5	185.6	86.6	391.4	682.4	776.3	128.7	133.1	1218.0
Case55	Died	1619.6	106.6	652.4	1022.3	776.2	366.9	734.3	515.3	1017.1
Case56	Survived	159.4	76.6	23.7	48.8	9135.9	633.5	46.2	24.2	426.6
Case57	Survived	776.8	204.4	1342.2	2300.3	1361.0	1931.6	616.1	568.0	1424.2
Case58	Died	1200.1	136.1	21.4	971.5	1295.9	830.9	26.2	16.0	1284.0
Case59	Died	1758.7	192.1	55.6	341.3	1662.4	2183.4	236.9	133.7	1194.7
Case60	Survived	1600.4	177.5	38.9	82.8	880.2	1934.4	101.4	93.8	619.1
Case61	Died	3001.0	109.3	992.9	2110.0	1907.6	330.5	1338.8	733.9	1380.2
Case62	Survived	1388.9	121.3	25.2	92.9	685.8	130.6	35.7	19.2	690.7
Case63	Survived	100.9	133.9	1606.2	3022.4	2323.8	1378.0	2800.2	736.4	1523.7
Case64	Survived	513.4	101.5	13.7	35.7	4005.7	105.3	31.4	19.8	283.1
Case65	Died	1942.8	122.8	3.4	74.0	1047.4	309.0	20.1	9.1	826.3
Case66	Survived	262.4	163.7	375.3	663.3	362.2	48.8	865.6	410.1	1318.8
Case67	Survived	750.2	111.3	29.0	516.7	11817.4	1117.3	51.5	33.7	1819.9
Case68	Survived	682.4	95.6	3.4	26.6	2598.3	754.5	44.4	24.2	568.8
Case69	Survived	1802.3	105.1	3.4	3.6	3300.5	219.8	22.7	9.1	418.5
Case70	Survived	266.9	53.8	3.4	3.6	239.4	141.4	20.9	9.1	396.8
Case71	Survived	177.7	103.5	3.4	3.6	4811.9	653.4	15.7	9.1	740.3
Case72	Survived	2783.0	160.9	3.4	53.8	2391.7	491.0	14.8	9.1	434.7
Case73	Survived	790.3	79.9	3.4	38.4	1377.2	875.1	33.1	19.8	362.6
Case74	Survived	598.1	122.7	35.9	84.1	30.5	510.9	66.0	63.4	455.3
Case75	Survived	593.2	98.1	61.7	110.5	4437.8	304.0	115.9	82.0	424.4
Case76	Survived	94.1	60.5	27.5	41.0	410.0	365.2	72.4	42.5	368.1
Case77	Survived	2608.4	83.5	16.0	401.0	1118.4	340.4	32.3	16.0	1253.8
Case78	Survived	284.1	105.8	3.4	13.5	3029.1	251.1	20.9	9.1	587.2
Case79	Survived	1947.2	115.2	22.9	61.2	1377.2	620.3	41.8	21.7	709.0
Case80	Survived	801.3	81.6	50000.0	50000.0	1677.8	937.2	50000.0	50000.0	7974.3
Case81	Died	591.2	58.1	3.4	13.8	8861.0	576.8	22.5	9.1	472.9
Case82	Survived	885.0	105.6	546.3	860.0	840.0	837.5	1625.4	720.2	927.9
Case83	Survived	1839.6	183.9	16.0	91.2	36889.7	747.9	53.0	69.8	1021.6
Case84	Died	288.3	123.8	28.2	1122.4	1008.4	2680.1	47.4	29.3	1690.2
Case85	Died	2886.2	365.8	165.5	499.7	242.6	867.6	182.3	113.1	563.1
Case86	Survived	1535.7	59.0	174.1	835.5	385.3	1240.4	471.2	257.2	1815.7
Case87	Survived	2758.0	152.8	3.4	910.9	7450.7	4571.4	19.1	9.1	664.7
Case88	Survived	445.9	75.8	1256.8	2690.7	68.8	357.1	1792.4	1181.5	1283.0
Case89	Survived	1518.8	110.4	3.4	3.6	321.9	768.5	20.2	9.1	365.7
Case90	Survived	1244.2	115.8	2716.1	3599.7	2287.5	2840.3	4936.7	2446.3	707.6
Case91	Survived	1555.3	140.7	112.4	135.0	551.6	1669.9	175.9	127.1	537.9
Case92	Survived	3001.0	213.8	3.4	47.2	314.0	1673.6	23.7	13.0	732.0
Case93	Survived	1299.2	73.7	34.7	60.5	5686.1	313.4	66.8	38.2	325.8
Case94	Survived	1573.4	98.9	27.2	53.2	1252.4	325.9	56.1	33.4	149.6
Case95	Survived	73.2	73.1	445.6	469.4	27723.4	846.9	680.7	302.9	391.9
Case96	Survived	2363.8	244.9	13768.3	19018.6	567.5	1519.2	4052.2	6412.9	2450.5

Case no	Outcome	SAA (mg/L)	CRP (mg/L)	IL-2 (pg/mL)	IL-6 (pg/mL)	IL-8 (pg/mL)	IL-10 (pg/mL)	IL-18 (pg/mL)	GM-CSF (pg/mL)	MCP-1 (pg/mL)
Case97	Survived	1337.9	132.0	14.1	102.9	804.6	140.0	39.7	18.7	501.9
Control1		8.9	1.3	12.2	42.4	1655.3	8.4	34.9	18.2	115.0
Control2		21.6	3.8	227.3	291.7	7898.4	401.3	851.9	387.7	278.9
Control3		11.1	2.6	4162.6	4393.4	1528.1	19.5	5241.0	2501.7	334.0
Control4		16.3	15.2	3.4	16.1	837.3	1015.8	15.7	16.6	20.9
Control5		9.2	1.9	16.0	43.6	2930.3	56.6	32.3	27.4	234.5
Control6		10.0	2.1	34.3	68.9	3769.1	280.9	43.6	21.1	208.6
Control7		15.0	5.5	13.1	23.4	4063.1	6612.4	24.8	9.1	174.2
Control8		20.2	1.8	405.5	663.1	4886.1	8.4	367.1	9.1	275.3
Control9		15.3	2.7	3.4	3.6	1291.1	8.4	5.7	9.1	94.5
Control10		12.3	2.1	12.2	26.5	6033.5	30.5	33.7	34.5	103.8
Control11		13.2	2.5	937.0	1355.5	1261.0	8.4	1393.5	783.3	270.7
Control12		14.2	4.6	19.0	29.7	5810.5	135.9	53.1	21.8	211.7
Control13		14.4	3.1	168.2	189.3	3998.5	95.5	264.1	166.3	321.2
Control14		14.0	2.2	54.0	348.3	3316.7	8.4	420.1	125.8	222.6
Control15		14.1	2.7	3.4	3.6	1767.2	44.6	5.7	9.1	160.3
